# Antivirals Targeting Coronavirus RNA‐Dependent RNA Polymerase and Main Protease: From Mechanisms of Action to Outcomes in COVID‐19 Clinical Trials

**DOI:** 10.1111/1751-7915.70342

**Published:** 2026-03-31

**Authors:** Harald Brüssow

**Affiliations:** ^1^ Department of Biosystems, Laboratory of Gene Technology KU Leuven Leuven Belgium

## Abstract

The rapid global spread of SARS‐CoV‐2 triggered an unprecedented effort to develop effective antivirals. Among the first approved agents was remdesivir, an injectable nucleoside analogue developed by Gilead Sciences, that led to chain termination of viral RNA synthesis and showed broad antiviral activity against RNA viruses. Early clinical results were mixed: The US ACTT‐1 trial reported an accelerated recovery and reduced mortality in treated patients, while the WHO Solidarity and a European trial revealed no impact of remdesivir on mortality. In contrast, a US trial in outpatients demonstrated a clear clinical benefit when treatment was administered early. Molnupiravir, an orally applicable nucleoside analogue developed by Merck, induces lethal mutations in the viral genome rather than chain termination. Molnupiravir showed in vivo antiviral activity against coronaviruses in different animals. In MOVe‐OUT trials, molnupiravir reduced the rate of hospitalisation in treated outpatients. In the PANORAMIC trial, molnupiravir reduced the time to recovery in outpatients but not their rate of hospitalisation. No drug effect of molnupiravir was seen in the RECOVERY trial with hospitalised COVID‐19 patients. Using structural biology and medicinal chemistry approaches, Pfizer developed nirmatrelvir, an oral inhibitor of the major coronavirus protease. In high‐risk but not in standard‐risk COVID‐19 patients, the combination nirmatrelvir/ritonavir reduced the rate of hospitalisation (EPIC HR and SR trials). Retrospective cohort studies showed treatment effects in defined patient groups. This review compares the efficacy and clinical performance of different antivirals, including emerging drugs such as obeldesivir and alternative protease inhibitors (lopinavir, simnotrelvir). It further examines their roles in prophylaxis, treatment of long covid symptoms, pharmacological considerations and antiviral resistance. Particular attention is given to factors underlying variable outcome of the trials, including viral variant evolution, population immunity increases, disease severity changes and timing of therapy initiation.

## Graphical abstract

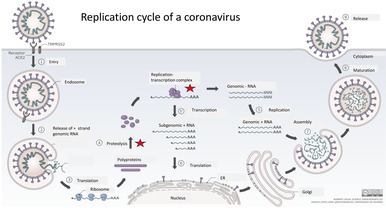

SARS‐CoV‐2 has become yesterday's news, ranking in public risk perception with flu or even common cold. Mortality and hospitalisation data indeed showed a drastic reduction. In January 2021, at the peak of the COVID‐19 epidemic in the US, 26,000 COVID‐19 deaths were counted nationally per week. This was a staggering figure when compared to the 58,000 US soldiers killed during the entire Vietnam war which traumatised the US society for years. During the 2024 summer wave of SARS‐CoV‐2 infection which deviated from recent declining trends, the death count in the US was 600 per week. This trend was also seen in hospitalisations. The hospitalisation rate for COVID‐19 in the US reached with the Omicron wave its peak in 2022 and amounted to 35 people hospitalised per 100,000 population per week. During the 2024 COVID‐19 summer wave this number was 4 (Cohen 2024). Some scientific forecasts see SARS‐CoV‐2 as joining the group of seasonal winter respiratory viral infections and parts of the public see this as a justification for a return to business as usual.

On this background, one might ask whether a review on COVID‐19 antivirals is a timely contribution. There are, however, a number of arguments why this view might be misleading. First, there are caveats from medical history. If the Russian flu pandemic of 1889 was a prior zoonotic coronavirus infection (Brüssow and Brüssow 2021)—and this is still a big if—it was followed 10 years later by a second clinically severe infection wave (Brüssow 2021). One can thus not exclude the possibility that SARS‐CoV‐2 might return with a more virulent variant in the future. Second, if not with a new SARS‐CoV‐2 variant, coronaviruses as a group are a fertile ground for new zoonotic infections spilling over into the human population as documented by the SARS epidemic of 2002/3, the Middle East Respiratory Syndrome (MERS) coronavirus emerging in 2012, followed by SARS‐CoV‐2 in 2019, documenting three distinct coronavirus epidemics within two decades. Third, potent antivirals—if effective—could be an important element of pandemic preparedness since several RNA viruses belonging to different viral families share sequence and structure‐related viral enzymes vital for their replication such as the RNA‐dependent RNA polymerase (RdRp) (Figure 1) or viral proteases. Broadly cross‐reacting small molecule viral inhibitors are therefore not an unrealistic goal (Lu et al., 2021) and would be valuable tools should a new RNA virus emerge. Fourth, there is an urgent need of drug treatment for post‐acute sequelae of SARS‐CoV‐infection (PASC) also called ‘long Covid’. For the US it has been estimated that about 7% of non‐institutionalised persons develop long Covid after an acute SARS‐CoV‐2 infection (Rosen 2024). The sheer number of persons affected in their quality of life by long Covid represent an enormous challenge for the health system even if acute COVID‐19 is now a lesser burden (Nikolich and Rosen 2023). The pathogenesis of long Covid is not yet clear (Greenhalgh et al. 2024) but there are indications that persistent viral infections might play a role in this condition and by consequence antivirals could play a role in its therapy (Al‐Aly 2025). For all these reasons, an assessment of the clinical effectiveness of approved coronavirus antivirals, addressed in the present review, is an important issue.

**FIGURE 1:**
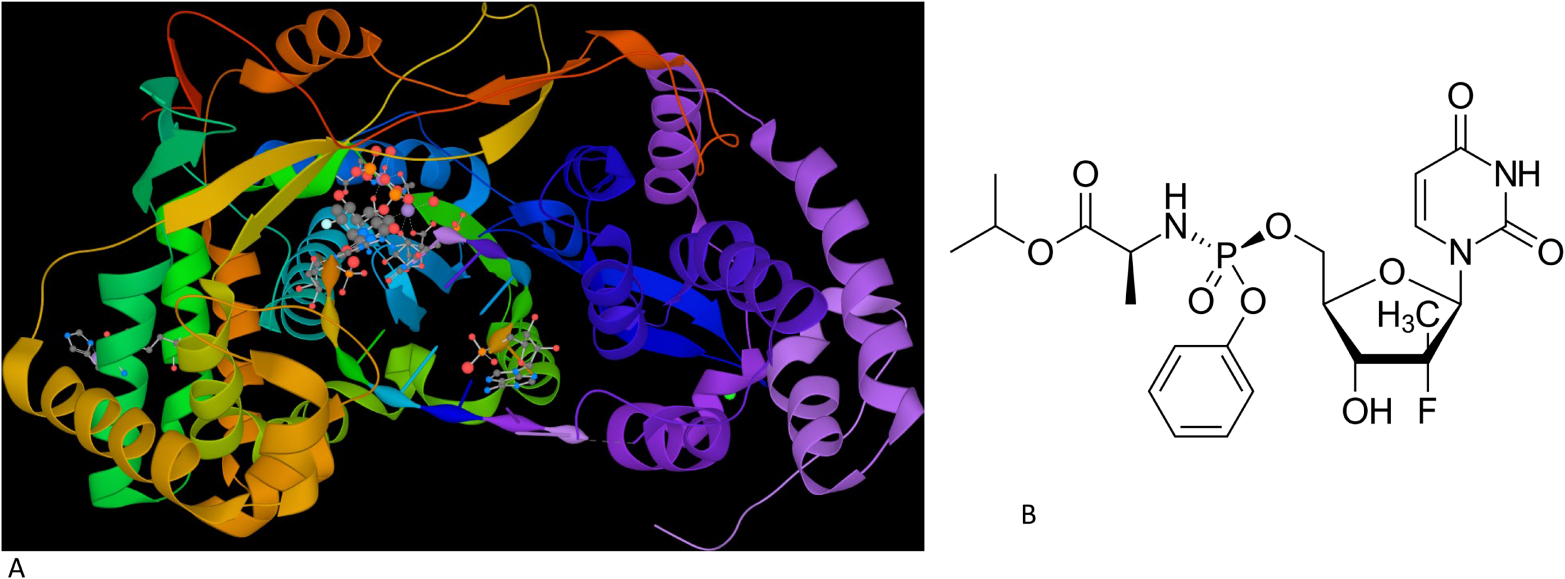
RNA replicase from RNA viruses and their inhibitors. Hepatitis C virus RNA replicase is shown here in complex with its inhibitor sofosbuvir. Molnupiravir, an approved inhibitor of SARS‐CoV‐2 RNA‐dependent RNA polymerase, was first described as an inhibitor of Hepatitis C virus, suggesting the conservation of structural elements in RNA replicases between different RNA viruses, which makes the development of antivirals with broad inhibitory activity a possibility. Figure credit: Artoria 2e5 from Wikipedia.

## 1 Remdesivir

### 1.1 Preclinical Work

**FIGURE 2:**
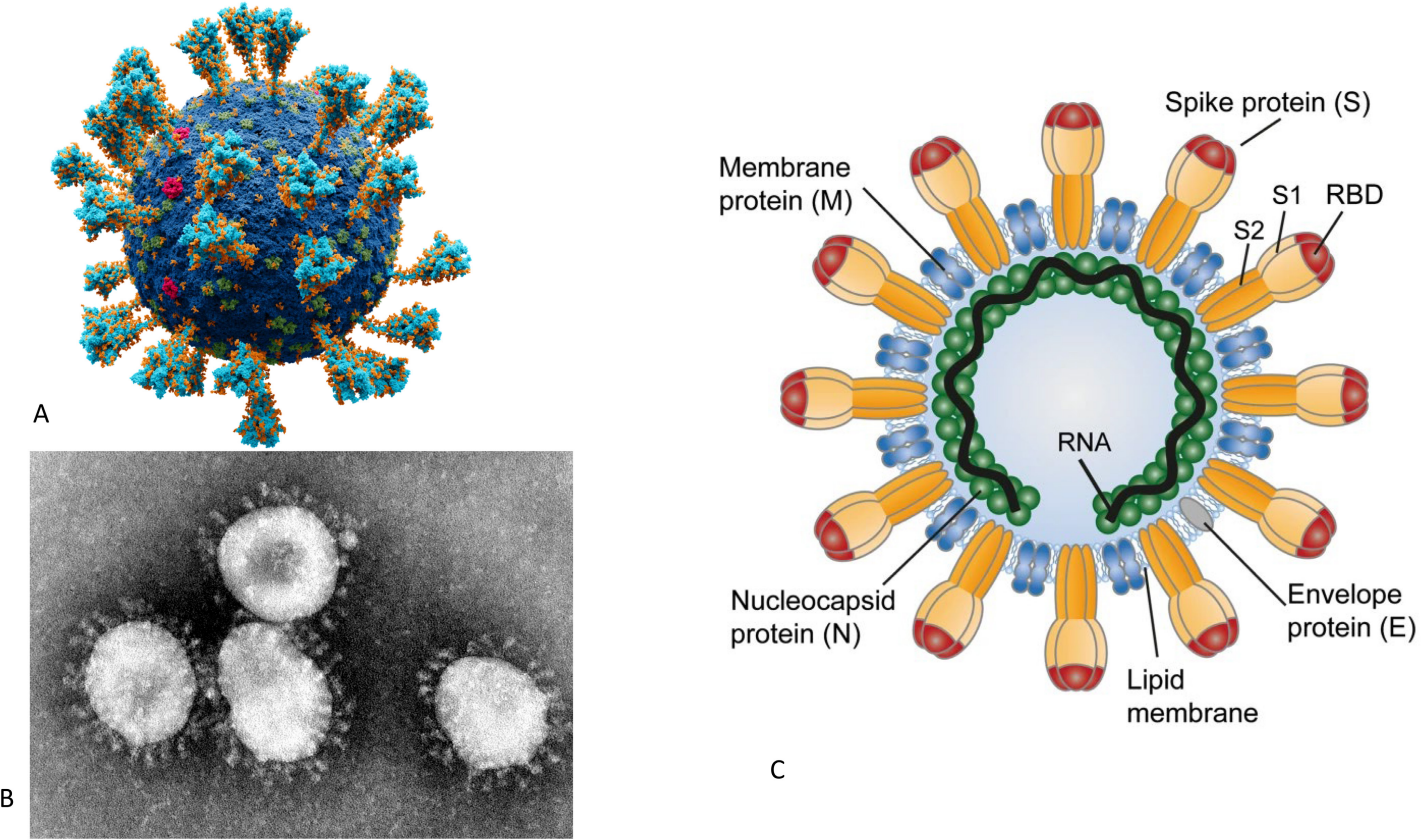
Coronavirus SARS‐CoV‐2 virion structure, genome map and encoded viral proteins. (A) Atomic model of the external structure of SARS‐CoV‐2. (B) Electron microscopy of human coronavirus 229E. (C) Genome map of SARS‐CoV‐2 indicating the open reading frames encoding the two viral non‐structural polyproteins and the individual non‐structural proteins Nsp 1 to Nsp16 released by proteolysis. Nsp5 is the main protease Mpro/3CL protease which releases Nsp5 to Nsp16. Nsp12 is the RNA‐dependent RNA polymerase. At the right side are the four structural proteins and accessory factors. Figure credit: (A) A. Solodovnikov & V. Arkhipova; (B) F. Murphy/CDC; (C) D. Gordon; all from Wikipedia.

**FIGURE 3:**
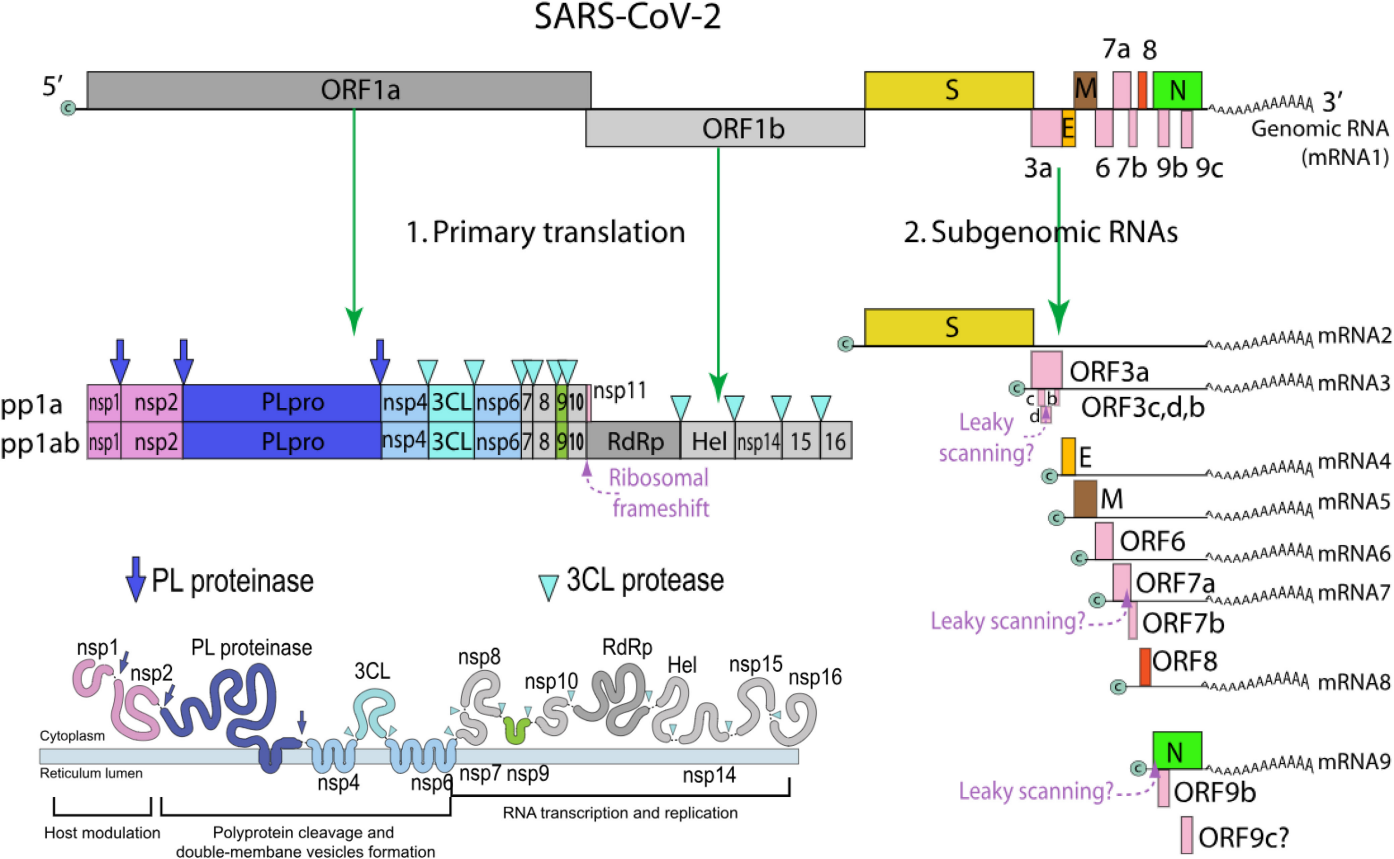
Genome map of SARS‐CoV‐2 with primary translation products for non‐structural proteins and subgenomic RNA products for structural proteins and additional genes. The proteolytic processing of the viral polyproteins by PL proteinase and 3CL protease is indicated in the two inserts. Figure credit: ViralZone, Swiss Institute of Bioinformatics (De Castro et al. 2024).

**FIGURE 4:**
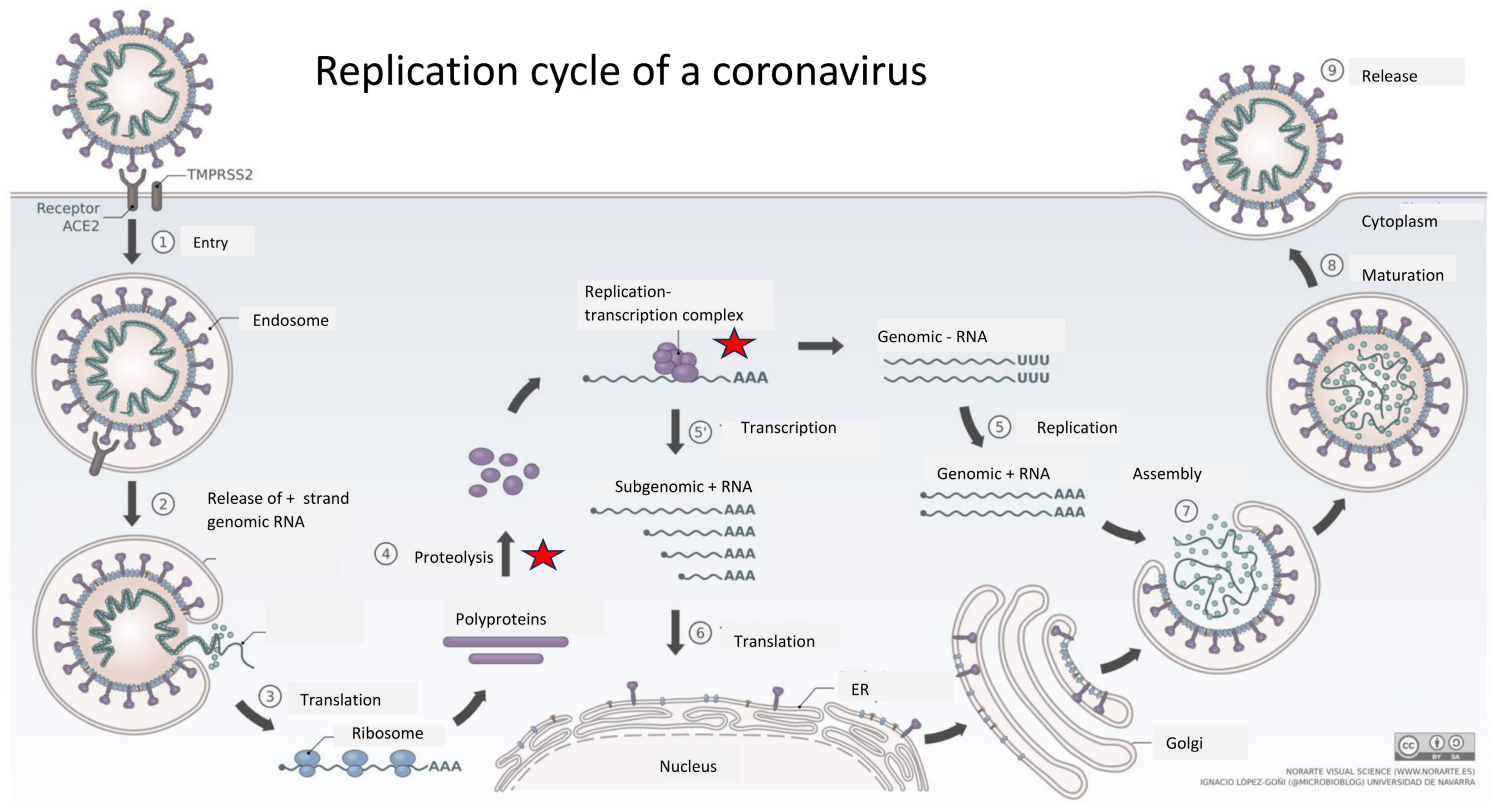
The replication cycle of SARS‐CoV‐2. The target sites of COVID‐19 antivirals discussed in the present opinion paper, namely the RNA dependent RNA polymerase Nsp12 and of the main viral protease Nsp5, are indicated by a red star in the overview of the replication cycle of coronavirus SARS‐CoV‐2. Figure credit: Vega Asensio from Wikipedia.

**FIGURE 5:**
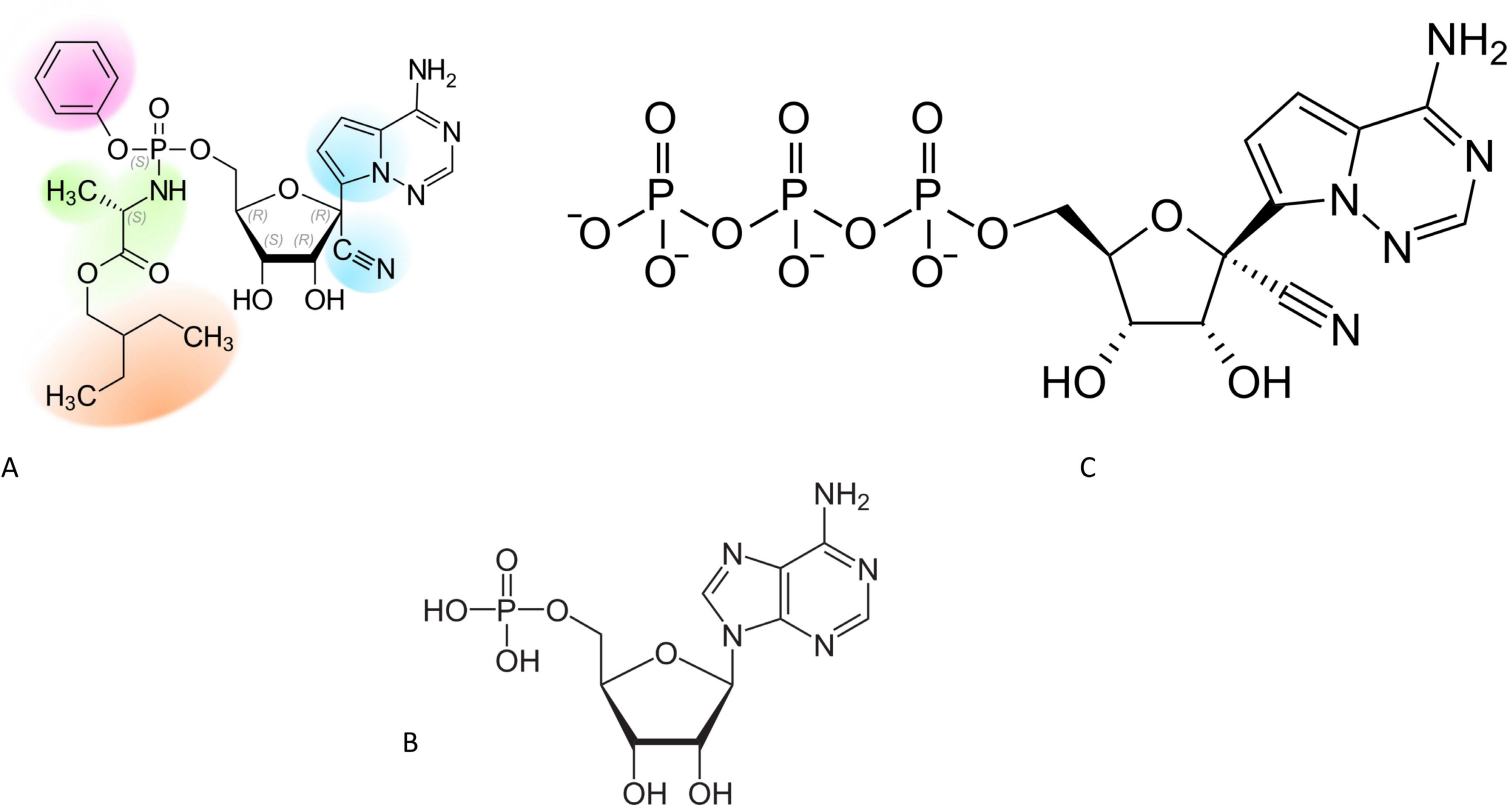
Remdesivir: A chain termination inhibitor of the RNA‐dependent RNA polymerase of SARS‐CoV‐2. Remdesivir (A) composition: Adenosine nucleotide analogue modified in the base (blue) by a changed 5‐ring heterocycle and the ribose (blue) by an addition of a cyano group and in the phosphate group by an addition of a phenyl group (violet), an alanine amino acid group (green) esterified to an ethylbutyryl group (orange). For comparison the natural AMP nucleotide is displayed (B). Remdesivir is metabolised and phosphorylated in vivo; the physiologically active form is shown in (C). Figure credit: Benff; Anypodetos both from Wikipedia.

Well ahead of the COVID‐19 pandemic, researchers argued that coronaviruses (Figure 2) represent a source of zoonotic infections with epidemic potential in humans. Scientists identified several conserved druggable enzyme targets in coronaviruses (CoV). This list included the 3C‐like protease (3CL or main protease M^pro^), the papain‐like (PL) protease, the nonstructural protein 12 (Nsp12, the RNA‐dependent RNA polymerase RdRp) (Figures 3 and 4). A collaboration between academic researchers and industrial scientists at Gilead Sciences Inc. developed a promising small molecule RdRp inhibitor active against various RNA viruses (filovirus, paramyxovirus, arenavirus and CoV). The inhibitor was an ethylbutyl alaninate phosphoramidate prodrug, GS‐5734, acting as a cyano‐modified adenosine analogue (Figure 5). Intracellularly, it was cleaved and then phosphorylated by host kinases to the triphosphate nucleotide. The viral RdRp accepted this analogue and incorporated it into the growing RNA strand, leading to chain termination. Neither the cellular nor the mitochondrial DNA‐dependent RNA polymerase accepted this analogue, explaining the absence of cytotoxicity. In rhesus macaques, the triphosphate had a half‐life of 14 h allowing a once‐per‐day drug dosing scheme. In a lethal infection model with ebolavirus, GS‐5734 (in later clinical tests it was called remdesivir) assured the survival of the monkeys when given 3 days after the viral challenge. Plasma viral titre was significantly decreased in treated animals and no resistance developed. GS‐5734 was amenable to large‐scale industrial production (Warren et al. 2016). Notably, remdesivir also protected African green monkeys against lethal infection with Nipahvirus, an emerging paramyxovirus, when given 1 day after virus challenge. Virus replication was not decreased in the upper respiratory tract of the animals, but viremia was suppressed (Lo et al. 2019).

The development of antiviral nucleosides against coronaviruses is complicated by the fact that CoV possess a proofreading 3′‐5′ exoribonuclease (ExoN). In fact, resistance of CoV to the widely used antiviral drug ribavirin is due to its removal from the catalytic site of RdRp by ExoN. Work with a model CoV demonstrated that a viral mutant lacking ExoN proofreading was indeed significantly more sensitive to GS‐5734. Mutants that conferred decreased sensitivity to GS‐5734 mapped to two amino acid positions in RdRp (Agostini et al. 2018). Surprisingly, the CoV RdRp showed a preference for the tri‐phosphorylated analogue over the natural nucleotide ATP. Due to the presence of a 3′‐OH group in the ribose of the analogue, GS‐5734 does not lead to an immediate chain termination in the synthesis of the new RNA strand. RNA synthesis only terminates after a few more nucleotide incorporation events (Gordon et al. 2020). Chinese researchers analysed the structure of the SARS‐CoV‐2 RdRp, both in the apo form and in complex with a 50‐base template‐primer RNA and remdesivir. The exact atomic interactions of the inhibitor with the catalytic site of the replication complex provided the stereochemical knowledge for the development of even more potent inhibitors than remdesivir (Yin et al. 2020).

Subsequently, US researchers demonstrated a broad in vitro activity of remdesivir against a wide range of human and bat coronaviruses. Remdesivir has a very short half‐life in mice, but when working with mutant mice that lack the drug‐metabolising enzyme carboxylesterase, prophylactic treatment with remdesivir ameliorated weight loss, reduced virus titre in the lung, reduced lung pathology and prevented airway resistance development. Therapeutic treatment with remdesivir given 1 day after the viral challenge still significantly ameliorated all these disease parameters. However, when given 2 days after viral challenge, remdesivir did not decrease disease severity or survival but still reduced viral lung titers (Sheahan et al. 2017). Remdesivir was then explored in rhesus macaques against MERS CoV infection. Prophylactic remdesivir treatment completely suppressed clinical signs, viral replication in the lungs and the development of lung pathology (interstitial pneumonia). Therapeutic treatment with remdesivir initiated 12 h after viral challenge reduced the clinical score, lung pathology and reduced the viral load in the lung (de Wit et al. 2020).

In early 2020, US researchers had established a SARS‐CoV‐2 infection model in rhesus macaques which developed a transient respiratory disease. Twelve hours after virus challenge when viral titers reached a peak value, they applied daily intravenous remdesivir or a vehicle. Clinical scores were significantly lower in remdesivir‐treated monkeys, radiography showed less lung lobe involvement and less lung infiltration, and the treated animals showed lower lung viral loads and infectious virus titers than vehicle‐treated infected monkeys. At necropsy, gross lung pathology and histopathology showed that interstitial pneumonia and histopathology were greatly attenuated in treated animals. However, treated animals continued to shed infectious virus from the upper respiratory tract, but drug‐resistant viral mutants were not detected (Williamson et al. 2020).

### 1.2 Clinical Trials

During the early phase of the pandemic (Spring 2020) 1060 patients mostly with severe COVID‐19 disease from North America and Europe were treated in the ACTT‐1 trial with intravenous remdesivir (200 mg loading dose, followed by 9 days of 100 mg remdesivir) or placebo. In the severe disease stratum, time to recovery was significant shorter in treated than in placebo recipients (11 vs. 18 days). However, no beneficial effect was seen in patients already receiving mechanical ventilation at enrollment. The beneficial effect of remdesivir was greater when given earlier, that is, within 10 days after symptom onset. Mortality in the remdesivir group was at day 15 of treatment significantly lower than in the placebo group (6.7% vs. 11.9%), but the difference in mortality narrowed at day 29 (11.4% vs. 15.2%). Serious adverse events occurred at comparable rates in treatment and control group (Beigel et al. 2020). Between February and March 2020, also clinicians from Hubei/China enrolled 237 adult COVID‐19 patients with radiologically confirmed severe pneumonia at a 2:1 ratio on remdesivir or placebo, starting 10 days after symptom onset. Remdesivir use was not associated with a difference in time to clinical improvement (21 vs. 23 days) and 28‐day mortality was similar (14% vs. 13%). Viral load decreased over time similarly in both groups. Viral control measures in Wuhan led to an unplanned stop of patient enrolment, decreasing the power of the trial to detect differences (Wang et al. 2020). In a further phase 3 trial 584 moderately ill COVID‐19 patients from US, Europe and Asia were enrolled between March and April 2020 and randomised on a 5 day or a 10 day course of remdesivir or standard care. The clinical endpoint was the clinical status of the patients at day 11. Those on the 5 day but not those on the 10 day remdesivir course showed a better clinical score than those on standard care, but the clinicians attributed an uncertain clinical importance to this difference (Spinner et al. 2020).

In another phase 3 trial from the US, Europe and Asia, a 5 day and a 10 day remdesivir course were compared in 397 hospitalised COVID‐19 patients with severe radiologically confirmed pneumonia. Clinical improvement on an ordinal scale occurred in 64% of patients in the 5‐day group and in 54% in the 10‐day group. Since the latter group had a worse baseline condition, the clinicians concluded that both treatments were of comparable efficacy. Since the trial was run without a placebo group, the magnitude of the clinical benefit could not be determined (Goldman et al. 2020). A compassionate treatment trial with remdesivir was conducted in February 2020 in 53 COVID‐19 patients, half of them receiving mechanical ventilation at baseline. Clinical improvement was observed in 68% of these severely affected patients; mortality was 13% (Grein et al. 2020).

An interim analysis of the WHO‐guided Solidarity trial of repurposed antiviral drugs (testing remdesivir, hydroxychloroquine, lopinavir and interferon beta‐1a or standard care) among 11,330 adults hospitalised with COVID‐19 showed little or no effect with respect to overall mortality, initiation of ventilation and duration of hospital stay. Specifically, death occurred in 301 of 2743 patients receiving remdesivir and in 303 of 2708 receiving standard care. Remdesivir did not reduce the initiation of mechanical ventilation among treated patients (295 vs. 284 in controls) (WHO Solidarity Trial Consortium 2021). The Solidarity trial was an adaptive trial where unpromising antiviral drugs were eliminated during its course. This happened to hydroxychloroquine, lopinavir and interferon beta‐1a. Remdesivir remained in the trial until donated supplies of remdesivir became low in February 2021. At this time point, 4146 patients had been randomised on remdesivir and 4129 on standard care (which included in 68% corticosteroids). In‐hospital mortality, the primary outcome, was seen in 602 and 643 patients, respectively; the difference was statistically not significant. Furthermore, 14% of patients assigned to remdesivir and 16% of patients assigned to control progressed to ventilation. Remdesivir treatment did not reduce time to discharge from the hospital (WHO Solidarity Trial Consortium 2022).

Until January 2021 the European DisCoVeRy trial enrolled 857 COVID‐19 patients who required oxygen supplementation on remdesivir or standard care. No significant difference in clinical status assessed on an ordinal scale was observed between the remdesivir and the control group at day 15 and 29, including subgroup analyses. The proportion of deaths at day 28 and at 3 months was not significantly different. The viral load did not differ between the two groups (Ader et al. 2022a). In a final analysis, less patients in the remdesivir group progressed to mechanical ventilation than controls (Ader et al. 2022b). In contrast, a clinical trial in Thailand following the decrease in viral oropharyngeal load in early symptomatic COVID‐19 patients observed an accelerated viral decrease in 67 remdesivir‐treated compared to 64 control patients receiving no drugs (Jittamala et al. 2023).

In the winter 2020/2021 US clinicians enrolled 562 adult symptomatic COVID‐19 outpatients into a 3‐day course of remdesivir treatment or placebo. The patients were mostly White, obese and relatively young (mean age 50 years). They had already experienced 5 days of symptoms when treatment started. Remdesivir treatment was associated with a lower rate of hospitalisation (0.7% vs. 5.3% in placebo), of COVID‐19–related medical visits (1.6% vs. 8.3%) within the next month; 35% of remdesivir treated outpatients reported an alleviation of symptoms by day 14 compared to 25% of the placebo recipients. However, remdesivir treatment had no effect on the decrease of the viral load compared to placebo (Gottlieb et al. 2022).

In the Finnish part of the SOLIDARITY trial, 208 patients were randomised on remdesivir or placebo. After a 1‐year follow‐up, no difference in mortality, in self‐reported recovery and in long Covid associated symptoms was observed between the two groups (Nevalainen et al. 2022).

### 1.3 Resistance

When SARS‐CoV‐2 was cultivated in vitro in the presence of remdesivir, a mutant virus was obtained that showed an amino acid change (E802D) in the RNA‐dependent RNA polymerase which conferred partial resistance to remdesivir without affecting the replication fitness of the mutant virus. However, this mutant was not identified in the SARS‐CoV‐2 genomic database from patients (Szemiel et al. 2021). An immunocompromised patient with an acquired B cell deficiency developed de novo this E802D mutation during remdesivir therapy and this was associated with a rebound of viral replication and a worsening of clinical symptoms, necessitating treatment with monoclonal antibodies (Gandhi et al. 2022). German researchers followed longitudinally 14 COVID‐19 patients who showed a persistent viral infection for up to 140 days without being immune‐compromised. Most patients showed a surprisingly stable viral genome sequence except for one patient who was treated with remdesivir (Heyer et al. 2022).

## 2 Molnupiravir

### 2.1 Preclinical Work

**FIGURE 6:**
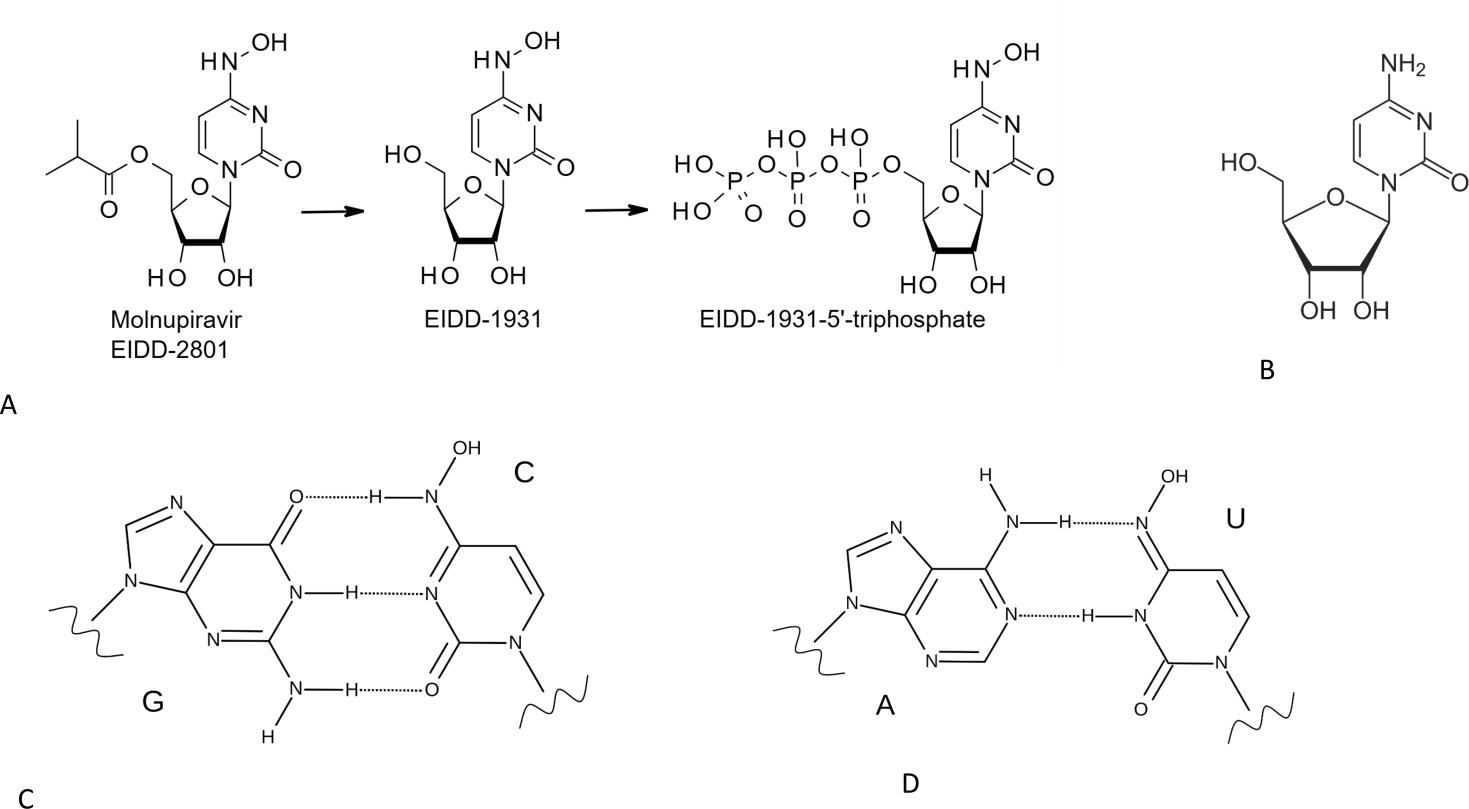
Molnupiravir: An RNA replicase inhibitor leading to error catastrophe. (A) Molnupiravir (left) is metabolised to a ribonucleoside nucleoside (centre) and is then further phosphorylated to the nucleotide triphosphate N‐hydroxy cytidine NHC‐TP (A right). EIDD‐1931is an analogue of the natural nucleoside cytidine (B). NHC can tautomerise between two forms which either bind to guanine base (C) or adenine base (D) introducing transition mutations in the viral RNA. *Source:* M D Turnbill from Wikipedia.

More than 20 years ago, researchers at Emory University (Atlanta/USA) observed that a base‐modified nucleoside analogue, N‐hydroxycytidine (NHC), inhibited RNA genome synthesis of bovine viral diarrhoea virus, a pestivirus and hepatitis C virus in cell culture assays. In vitro biochemical assays showed that synthetic NHC‐triphosphate (NHC‐TP) did not inhibit the polymerisation reaction by the viral RNA‐dependent RNA polymerase and the researchers suspected that NHC‐TP served as a substrate for the viral enzyme. Upon serial cell culture passage of the pestivirus, resistance development was not observed (Stuyver et al. 2003). In 2018, these US researchers demonstrated a strong reduction of infectious titre for Venezuelan equine encephalitis virus, an alphavirus (Togaviridae) when grown in cell culture in the presence of NHC. They observed a 10‐fold increase of the mutation rate of the viral RNA genome after a single cell culture passage (Urakova et al. 2018). Encouraged by these results the Emory researchers extended their research to another virus of major medical importance, influenza A virus. NHC had a good oral bioavailability in rodents, but in primates and ferrets, the best animal model for human influenza virus infections, an isopropylester‐derivative of NHC had to be developed as a suitable oral prodrug (Figure 6A). In vivo a widely distributed carboxylesterase cleaves the isopropyl rest during adsorption or during first hepatic pass since in blood only NHC was observed. Circulating NHC is then taken up into cells via multiple nucleoside uptake transporters. Intracellularly, NHC is phosphorylated by host kinases to NHC‐TP and because of the permissiveness of the viral RNA‐dependent RNA polymerase, NHC becomes incorporated into the elongating RNA strand. Due to its ability to tautomerise, NHC can substitute for either cytidine or uridine and then pair with either guanosine or adenosine during subsequent viral RNA replication, causing multiple mutations (Maas et al. 2024) (Figure 6C,D). In ferrets, the prodrug was influenza A virus sterilising when given prophylactically or post‐exposure. It reduced nasal viral titers by four logs when given therapeutically at the moment of symptoms appearance. Virus was not detected in the lungs by immunohistochemistry in treated animals and the duration of fever was reduced. Resistance development was not observed in ferrets. In addition, off‐target mutations were not seen in host mRNA when using viral sterilising prodrug doses (Toots et al. 2019). NHC showed antiviral activity against other RNA viruses including coronaviruses, which are unusual among RNA viruses in displaying a proof‐reading activity during RNA replication. In the presence of NHC, coronaviral RNA levels were reduced 10‐fold but viral infectivity decreased 5000‐fold. The viral genome showed transition mutations; their rate increased with NHC concentration. Upon serial passage in the presence of NHC, viruses showed mutations scattered through the genome but only minimal levels of drug resistance (Agostini et al. 2019). With the onset of the COVID‐19 pandemic the US researchers extended their work to a wider range of coronaviruses containing three human viruses (MERS, SARS‐CoV, SARS‐CoV‐2). They observed again a dose dependent reduction of viral titers with maximal inhibition well below cytotoxicity levels of NHC in cell culture. NHC was active against SARS‐CoV‐2 in primary human airway epithelia, inhibited SARS‐CoV‐2 isolates that were resistant against the antiviral remdesivir and showed inhibitory activity against three zoonotic bat coronaviruses. In the presence of 1 μM NHC, MERS CoV showed upon RNA genome sequencing a 5‐fold increase in mutation rate and a 140‐fold decrease in virus titre. At 10 μM NHC, virus titre decreased by 26,000‐fold. In mice infected with SARS‐CoV, NHC reduced weight loss, lung haemorrhage and lung viral titre compared to vehicle‐treated controls. Under therapeutic treatment weight loss and lung haemorrhage were significantly diminished when NHC was given up to 24 h after viral challenge. Similar inhibitory effects were seen for NHC in mice challenged with MERS CoV. RNA sequencing revealed a mutation rate of 8 errors per 10,000 viral bases; a fifth led to amino acid changes suggesting an error catastrophe–driven mechanism of action for NHC and not chain termination. Cellular mRNA did not show mutations in NHC‐treated infected mice (Sheahan et al. 2020). Ferrets demonstrate a productive SARS‐CoV‐2 replication in the upper respiratory tract but show only low viral lung titers and a mild self‐limiting disease. SARS‐CoV‐2 infected ferrets transmit the infection efficiently to co‐housed control ferrets, presenting an attractive model for viral transmission to contact persons. Notably, molnupiravir given at a dose of 5 mg/kg body weight starting 12 h after viral challenge prevented viral infection transmission to co‐housed ferrets. No viral RNA was detected in the co‐housed ferrets while infected ferrets not receiving molnupiravir infected all sentinel ferrets (Cox et al. 2021). Other US researchers confirmed that molnupiravir treated ferrets showed reduced nasal viral shedding compared to controls irrespective whether ferrets were infected with SARS‐CoV‐2 alpha, beta, gamma or delta variant. Treatment suppressed the transmission of all variants to sentinel ferrets (omicron did not transmit). Since ferrets do not develop lung disease upon infection, the researchers used dwarf hamsters as model that showed severe signs of disease (weight loss, hypothermia, lethargy, dyspnea, lung pathology) when infected with the different SARS‐CoV‐2 variants of concern. The pathogenicity varied between the variants (alpha lowest, delta highest). Treatment with molnupiravir starting 12 h after infection when high viral lung titers were observed alleviated symptoms, reduced viral lung titers by 1 (delta) to 4 orders of magnitude (gamma). Notably, lung titre reduction was statistically significant in male, but not female hamsters (Lieber et al. 2022).

### 2.2 Clinical Trials

In a phase 1 clinical trial, healthy human subjects from UK received oral NHC (called molnupiravir in clinical development by Merck) once or twice per day in doses increasing from 50 to 1600 mg or placebo. NHC appeared rapidly in plasma, reached peak titers after 1.5 h and showed a half life of 1 h. No serious adverse events were observed (Painter et al. 2021). In a phase 2 clinical trial, 202 SARS‐CoV‐2 outpatients received within 7 days after symptom onset either 200, 400 or 800 mg molnupiravir twice daily or placebo. Viral load was assessed by RT‐PCR on nasal swabs and infectious virus was tested in cell culture. Time to viral RNA clearance was 1 day shorter only in the 800 mg molnupiravir group compared to placebo. Molnupiravir had no significant effect on the resolution of clinical symptoms (Fischer 2nd et al. 2022). In the subsequent phase 2 MOVe‐OUT trial, 302 outpatients with mild to moderate laboratory confirmed COVID‐19 were treated. The primary endpoint was the rate of hospitalisation or death (3.1% vs. 5.4% in placebo recipients). A treatment effect was greater in the prespecified subgroup of patients older than 60 years (3.6% vs. 21.4% in controls). Viral RNA load reduction was seen in a dose‐dependent way only when treatment was started within 5 days of symptom onset (Caraco et al. 2022). In a phase 2/3 trial, 304 patients hospitalised with COVID‐19 displaying risk factors for severe disease received molnupiravir within 10 days after symptom onset. Median time to sustained recovery was 9 days in treated and placebo recipients and no difference in all‐cause mortality was seen. Viral error rate was higher in the 800 mg treatment than in the placebo group, but no difference in viral load reduction was seen compared to the placebo group (Arribas et al. 2022). In the phase 3 MOVe‐OUT trial 1433 nonhospitalised, unvaccinated adults with mild‐to‐moderate, laboratory‐confirmed COVID‐19 were treated with 800 mg molnupiravir or placebo within 5 days after symptom onset. The primary efficacy end point was the incidence of hospitalisation or death at day 29. In an interim analysis with half of the enrolled adults, molnupiravir treatment was associated with a significant lower endpoint rate (7.3% vs. 14.1% in placebo group). In the final analysis, the endpoint was still different, but the difference became smaller (6.8% vs. 9.7%). One death occurred in the treatment and 9 in the placebo group. Treatment compared to placebo was associated with a greater reduction of viral load from baseline. The erosion of the efficacy observed between the interim and final analysis was explained by the inclusion of new countries into the trial and changed patient characteristics (Jayk Bernal et al. 2022). For most COVID‐19 symptoms in this study (namely fatigue, shortness of breath, loss of smell), sustained resolution or improvement was more likely in the molnupiravir than in the placebo group (Guan et al. 2023). The UK‐based PANORAMIC open label prospective clinical trial randomised 25,000 vaccinated outpatients who were treated in 2022 with 800 mg molnupiravir plus standard care or standard care alone within 5 days of symptom onset. The primary outcome was all‐cause hospitalisation or death within 28 days which occurred in 1% of the molnupiravir and 1% of the standard care group. Median time from randomisation to first recovery was 9 days in the molnupiravir vs. 15 days in the usual care group. Molnupiravir treatment was associated with a tenfold viral load reduction compared to standard care (Butler et al. 2023). An observational study from the first half of 2022 in Hong Kong investigated the effectiveness of molnupiravir under real world conditions: 4900 COVID‐19 patients were treated with 800 mg molnupiravir and compared with matched 49,000 untreated patients when omicron was the dominant pandemic SARS‐CoV‐2 variant. Molnupiravir treatment had no effect on the hospitalisation rate but significantly reduced in‐hospital disease progression (hazard ratio 0.57) and all‐cause mortality (hazard ratio 0.76) (Wong et al. 2022). From data of a national healthcare provider in Israel during the omicron wave of the pandemic, researchers matched 2600 subjects who received molnupiravir mostly within 3 days after a positive SARS‐CoV‐2 test to 2600 controls that did not receive antivirals. Molnupiravir treatment was associated with a non‐significant reduction of severe COVID‐19 outcome or death. Subgroup analysis suggested a risk reduction in older patients, females and those with inadequate vaccination (Najjar‐Debbiny et al. 2023). A small RCT was conducted in UK among 180 COVID‐19 patients with mild to moderate disease status. Recipients of molnupiravir had a significantly faster median time from randomisation to PCR negativity than controls (8 vs. 11 days), which was the primary endpoint of the trial (Khoo et al. 2023). Between 2022 and 2023 about 900 hospitalised COVID‐19 patients with pneumonia from UK, Nepal and Indonesia were treated in the RECOVERY trial format with molnupiravir or usual care (which included the antiviral remdesivir). The primary outcome was 28‐day mortality which was with 17% high, but did not differ between the two trial arms. Secondary endpoints were time to discharge from hospital and progress to invasive ventilation which did not differ between the two groups. The molnupiravir group showed lower nasal viral load than the control patients at 5 days, but not at 3 days after treatment start (RECOVERY Collaborative Group 2025).

### 2.3 Concerns

In general, SARS‐CoV‐2 evolved gradually during the pandemic. However, some variants such as alpha, omicron or BA.2.86 showed many mutational changes without that intermediates in their evolution could be identified in the viral genome database. Researchers suspected cryptic evolution for them in immunocompromised patients who experienced chronic infections (Kosakovsky Pond and Martin 2023). Sanderson et al. (2023) proposed an alternative hypothesis when analysing mutation patterns in millions of SARS‐CoV‐2 genome sequences from the database. Starting in 2022, they observed in phylogenetic trees long viral genome branches with high G‐to‐A transitions in countries with high molnupiravir prescription rates. Viruses had accumulated up to 100 mutations and they observed potential transmission clusters among patients. The density of transition mutations was particularly high in the gene encoding the viral spike protein suggesting positive immune selection. The researchers suggested that molnupiravir treatment has left a visible trace in global databases of SARS‐CoV‐2 genomes possibly leading to an accelerated viral evolution. While they do not link this with the emergence of variant viruses of concern, they expressed the concern that an antiviral might open viruses new genome evolution possibilities.

## 3 Nirmatrelvir

### 3.1 Preclinical Studies

**FIGURE 7:**
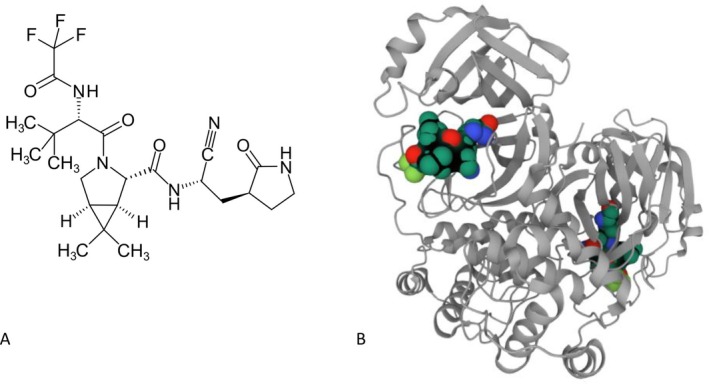
Nirmatrelvir, an inhibitor of the major protease from SARS‐CoV‐2. Nirmatrelvir (A) binds to the SARS‐CoV‐2 3CL protease dimer (B). The nitrile group of the drug represents the chemical warhead that reacts with the cysteine group in the catalytic site of the enzyme, inhibiting the proteolytic activity. *Source:* NadirSH, Next2u from Wikipedia.

Medicinal chemists from Pfizer had developed a small compound inhibitory drug directed against the main protease of SARS‐CoV causing the 2002 SARS outbreak. This compound also showed inhibitory activity against the enzymatic activity of 3CL (chymotrypsin‐like) protease of SARS‐CoV‐2, also known as M^pro^ main protease, which proteolytically cleaves the two viral polyproteins at 11 different sites to yield shorter non‐structural proteins essential for viral replication (Hoffman et al. 2020) (Figure 3). By co‐crystal structural analysis of inhibitor binding to the protease combined with stepwise medicinal chemistry modification of the compound, the Pfizer researchers developed a compound that showed increased antiviral activity in Vero cells at sub‐micromolar concentrations, good oral bioavailability, no off‐target activity against human protease (cysteine proteases which cut after Gln are not known in humans), and pan‐coronavirus inhibitory activity (Owen et al. 2021) (Figure 7). In a mouse SARS‐CoV‐2 infection model, the compound later called in clinical trials nirmatrelvir prevented infection‐induced weight loss, reduced in a dose‐dependent way the viral lung titre (up to 100‐fold), and lung histopathology. Pharmacological experiments in monkeys showed a first‐pass effect by cytochrome P450 reducing the plasma level of the drug. In a human phase 1 trial, nirmatrelvir was therefore combined with the P450 inhibitor ritonavir, which boosted the drug plasma level above the in vitro determined antiviral concentration for 12 h, allowing twice daily drug dosing.

### 3.2 Clinical Studies

The phase 2/3 EPIC‐HR trial was conducted worldwide in 2246 COVID‐19 patients at high risk of progression to severe disease; risk factors were obesity, smoking or hypertension. Half of the patients were treated twice‐daily with 300 mg of nirmatrelvir/100 mg ritonavir or placebo for 5 days; treatment started within 5 days of symptom onset. At interim analysis with half of the patients, treatment was associated with a 10‐fold reduction of hospitalisation or death compared to placebo (0.8% vs. 7%). A similar difference was seen in final analysis with all patients indicating a 89% relative risk reduction; 9 deaths occurred in the placebo and none in the treatment group. A consistent treatment effect was seen across all subgroups; more substantial treatment effects were seen in patients older than 65 years and in patients seronegative at baseline. Notably, the patients were enrolled into this study when the delta variant of SARS‐CoV‐2 was the main circulating virus and all patients were unvaccinated. Compared to controls a significant, but less than 10‐fold viral load reduction was seen in the treated patients. Adverse events were not more frequent in the treatment than in the placebo group (Hammond et al. 2022). A subsequent analysis of the EPIC‐HR data reported a 3‐day reduction in time to symptom resolution in treated as compared to placebo patients, a reduced rate of health care use and a shorter hospital stays, less intensive care use and less mortality in treated versus control patients who were hospitalised (Hammond et al. 2025). Researchers from Israel evaluated retrospectively health data from a national insurance company for 110,000 COVID‐19 outpatients during the omicron viral wave of the pandemic; 3900 of them received nirmatrelvir. Among 42,000 patients older than 65 year, hospitalisation due to COVID‐19 occurred in 15 treated cases compared to 59 untreated cases per 100,000 person‐years suggesting a four‐fold risk reduction. Death due to COVID‐19 was reduced 5‐fold in treated patients. A protective effect was seen in patients with and without preexisting immunity at baseline. In contrast, no nirmatrelvir treatment effect was detected among the 66,000 outpatients aged 40 to 65 years (Arbel et al. 2022). A retrospective cohort study of 20,000 non‐hospitalised adult patients infected with SARS‐CoV‐2 omicron variants during 2022 in Colorado showed an treatment effect of nirmatrelvir‐ritonavir vs. placebo. All‐cause hospitalisation in the month following infection was halved and mortality was also lower (2 vs. 15 deaths). Protection by treatment was extended to patients younger than 65 y (Aggarwal et al. 2023).

Data extraction from Californian electronic health records in 2022 identified 7000 nirmatrelvir/ritonavir treated patients and 126,000 non‐recipients among SARS‐CoV‐2 test‐positive persons in a setting with high vaccine uptake. The primary endpoint of hospital admission in the following month occurred in 0.7% and 0.6% of infected subjects, respectively. After adjustment for differences between the two groups, treatment was associated with a 54% reduction in hospitalisation rate which was increased to 80% if treatment was started within 5 days of positive SARS‐CoV‐2 tests (Lewnard et al. 2023). Evaluation of a health care register in New England states during the omicron infection wave showed that the hospitalisation rate in outpatients within two weeks after a positive SARS‐CoV‐2 test was low (< 1%), but nirmatrelvir/ritonavir treatment still halved this risk. The drug effect was greater in subjects that were not fully vaccinated (Dryden‐Peterson et al. 2023). An observational study from 2022 conducted in Hong Kong with 6400 COVID‐19 patients treated with nirmatrelvir plus ritonavir compared to matched controls associated a lower risk of death (hazard ratio HR of 0.34), hospitalisation (HR 0.76) and in‐hospital disease progression (HR 0.57) with drug treatment (Wong et al. 2022). In 2022, the Pfizer scientists conducted the phase 2/3 EPIC‐SR trial in COVID‐19 patients with standard risk (vaccinated subjects with a risk factor or unvaccinated subjects without risk factor). A total of 1288 patients were randomised to nirmatrelvir/ritonavir or placebo. Time to the alleviation of symptoms was 12 and 13 days in the treatment and placebo group, respectively. The rate of hospitalisation was low in both groups with 0.8% and 1.6%, respectively, and statistically not different. The main adverse effect associated with treatment was‐ as in the prior EPIC‐HR trial‐ dysgeusia (taste disturbance) in 6% of the treated patients. The Pfizer scientists concluded that nirmatrelvir treatment is of no proven use in low risk COVID‐19 patients (Hammond, Fountaine, et al. 2024). The Pfizer researchers led in parallel a phase 2/3 postexposure prophylaxis trial. They enrolled 2736 household contacts of COVID‐19 patients that were RT‐PCR negative for SARS‐CoV‐2 at baseline. The participants received 300 mg nirmatrelvir plus 100 mg ritonavir for 5 or 10 days or placebo. The primary endpoint was symptomatic COVID‐19 in the next 2 weeks which occurred in 2.6%, 2.4% and 3.9% of cases, respectively. Risk reduction by treatment was about 30% which was statistically not significant and did not meet the primary efficacy objective. Asymptomatic SARS‐CoV‐2 infection was reduced by nirmatrelvir. Viral load was reduced by 30‐fold with prophylactic treatment. The household transmission rate was low during this predominantly omicron infection wave which the authors attributed to seropositivity in 91% of the participants at baseline (Hammond, Yunis, et al. 2024).

Immuno‐compromised patients frequently excrete virus beyond the standard 5‐day treatment course. Pfizer scientists initiated a phase 2 trial in immunocompromised COVID‐19 patients comparing the effect of a 5, 10 and 15 day nirmatrelvir/ritonavir treatment on suppression of nasopharyngeal virus excretion by days 15 to 44. The endpoint was reached in 62%, 71% and 66% of patients, which was not statistically different between the treatment times. The 5 day treatment showed a longer median time to virus negativity in the subgroup of severely immunocompromised patients (Weinstein et al. 2025).

### 3.3 Resistance

US researchers cultivated SARS‐CoV‐2 in the presence of increasing concentrations of nirmatrelvir over multiple passages in cell lines. Up to a 50‐fold resistance increase was observed. Sequencing indicated a complex pattern of mutations in the 3CL protease suggesting multiple pathways to resistance. The researchers constructed single site mutations and associated a single site with high resistance (E166V with 100‐fold increase). Accompanying mutations compensated for fitness loss in viruses carrying this mutation. E166 mediates both nirmatrelsevir binding and 3CL protease dimerisation which is necessary for proteolytic activity. A lower degree of cross‐resistance was also observed in these mutants to ensitrelvir, an alternative inhibitor, binding to the same enzyme site but in a different mode. The identified mutations were also observed to circulate in the human population but at low frequency (Iketani et al. 2023). Pfizer scientists identified six mutation pattern that conferred > 20‐fold resistance to nirmatrelvir. E166V was the only emergent resistance mutation observed in three treated high risk patients (Zhu et al. 2024). Viral rebound without detectable mutations have also been reported in several nirmatrelvir‐treated patients (Charness et al. 2022).

## 4 Comparative Antiviral Trials

In the RECOVERY adaptive trial, COVID‐19 patients hospitalised mainly in the UK between 2022 and 2023 were treated with either molnupiravir or usual care (about 450 patients per group) or nirmatrelvir plus ritonavir or usual care (about 70 patients per group). The omicron variant was the infecting virus in all patients. In the molnupiravir as in the usual care arm, 17% of the patients died within 28 days. At day 5, but not at day 3 of molnupiravir treatment, a lower viral load was seen than under usual care. In the nirmatrelvir/ritonavir arm as well as the usual care arm, 19% of patients died. Viral load was lower at day 5 in the nirmatrelvir compared to the control group. In neither drug arm the researchers detected a difference in the duration of hospitalisation or the proportion of participants progressing to invasive ventilation. The low recruitment to the nirmatrelvir part precludes a definitive conclusion on its efficacy (RECOVERY Collaborative Group 2025). In Thailand, 209 young adults with low‐risk symptomatic COVID‐19 were in parallel randomised on molnupiravir, nirmatrelvir/ritonavir or no antiviral. Viral clearance half‐lives were 9 h with ritonavir‐boosted nirmatrelvir, 12 h with molnupiravir and 16 h in the controls. Nirmatrelvir and molnupiravir treatment reduced the viral load by 100‐fold and 10‐fold, respectively, compared to no drug treatment. Viral rebound was more frequently observed with nirmatrelvir than molnupiravir (10% vs. 2%); the latter showed an increased rate of transition mutations. Other antivirals were also tested in a prior enrolment phase of the Thai trial: favipiravir showed no impact on the viral clearance rate, while remdesivir showed an effect comparable to molnupiravir (Schilling et al. 2024). In another clinical trial conducted between 2023 and 2024 in Thailand, 600 low‐risk adults with early symptomatic COVID‐19 were randomised on nirmatrelvir/ritonavir or the alternative viral protease inhibitor ensitrelvir or no drug treatment. Both nirmatrelvir/ritonavir and ensitrelvir without ritonavir accelerated the viral clearance by a factor of two compared to the control group; no significant difference was observed between the two viral protease inhibitors. Clinical symptom resolution and viral rebound (7%) were comparable in all three groups (Schilling et al. 2026). A phase 2/3 clinical trial conducted in Japan during the Omicron epidemic in 340 subjects with mild to moderate COVID‐19 disease showed a significant but modest effect of ensitrelvir on viral clearance compared to a control group, but no significant effect on symptom resolution (Mukae et al. 2023).

Chinese clinicians compared nirmatrelvir‐ritonavir with VV116 treatment, a remdesivir hydrobromide derivative that showed, in contrast to remdesivir, good oral bioavailability. Overall, 380 mild COVID‐19 patients were randomised per treatment arm during the omicron infection wave in Shanghai, China. Despite risk factors (older age, hypertension, obesity), full recovery was achieved in 98% of the participants; sustained recovery was seen after a median of 4 and 5 days, respectively, in the VV116 and nirmatrelvir groups. Time from randomisation to a first negative SARS‐CoV‐2 test was 7 days in both groups. The VV116 group reported fewer adverse events than the nirmatrelvir group (Cao et al. 2023).

## 5 Antivirals Against Long Covid

Several retrospective cohort studies explored the impact of antiviral treatment in acute COVID‐19 patients on the later occurrence of post‐acute sequelae of SARS‐CoV‐infection (PASC). In 2022 the US Department of Veterans Affairs registered 229,000 subjects with a SARS‐CoV‐2 diagnostic; 11,500 were treated with molnupiravir within 5 days of a positive test and the rest received no antiviral. In this cohort study, risk for post‐acute sequelae was moderately reduced by antiviral treatment of the acute infection from 22% to 19%; post‐acute mortality was decreased from 2.3% to 1.4%, and post‐acute hospital admission from 10% to 9% when assessed half a year later. The treatment effect was statistically significant for fatigue, muscle pain, kidney injury and neurocognitive impairment, but risk reduction for these specific symptoms was small (about 1%). The treatment effect was independent from vaccination status (Xie et al. 2023b). In the same cohort study, Xie et al. (2023a) also analysed the impact of nirmatrelvir/ritonavir treatment given to 36,000 acute COVID‐19 patients compared to 246,000 controls not receiving antivirals. Read‐out was the rate of post Covid conditions assessed half a year later. Treatment caused a significant decrease in these symptoms from 17% to 13%, reduced hospitalisation from 7.5% to 6% and death from 1.4% to 0.7% (Xie et al. 2023a). Among nearly 4 million COVID‐19 outpatients older than 65 y from a US Medicare retrospective cohort study, 20% and 3% were treated in the acute phase with nirmatrelvir/ritonavir or molnupiravir, respectively. Absolute risk reduction for occurrence of PASC was statistically significant by both treatments, but small (2.7% for nirmatrelvir and 0.8% for molnupiravir) (Fung et al. 2023).

Comparable studies were also run in China. A retrospective real world cohort study was conducted with COVID‐19 patients hospitalised between 2022 and 2023 in Hong Kong. About 15,000 patients received directly after hospitalisation nirmatrelvir/ritonavir; 24,000 patients not receiving antivirals served as control. The outcome on 13 different post‐acute symptoms was assessed during a 1‐year follow‐up. Treated patients showed a significantly reduced mortality as well as significantly reduced rates for cardiological, pulmonary and renal diseases (Wang et al. 2024). In another large observational study from Hong Kong, 94,000 COVID‐19 patients received nirmatrelvir/ritonavir and 33,000 received molnupiravir during the acute infection. The long term outcome over 1 year was assessed and compared to 100,000 patients not receiving antivirals. Nirmatrelvir/ritonavir, but not molnupiravir, reduced mortality and hospitalisation over the 1 year follow‐up, but the effect size was small (Wei et al. 2025). In a third observational study from Hong Kong, half of 38,000 COVID‐19 patients received nirmatrelvir/ritonavir while the other half received no antivirals. After 1 year of follow‐up, the subjects were investigated for post‐acute health outcomes. Early treatment after hospitalisation reduced post‐acute all‐cause mortality and hospitalisation compared to the control group, while treatment starting 2 days or later after hospitalisation showed no effect (Chong et al. 2025).

Randomised prospective clinical trials in the context of long covid are rare. The patients of the UK‐based PANORAMIC trial treated with molnupiravir in the acute infection phase were also analysed for symptoms 3 and 6 months after the acute infection. Participants of the molnupiravir group felt better, experienced fewer and less severe persisting COVID‐19 associated symptoms, had fewer health consultations, and less missing from work than the control group, but the absolute difference between the groups was small (Harris et al. 2025).

Published randomised treatment trials of long covid patients with antivirals are so far scarce. In the PAX LC phase 2 clinical trial, 100 subjects with a proven SARS‐CoV‐2 infection and displaying long covid symptoms for at least 12 weeks were randomised at several US centres on nirmatrelvir/ritonavir or placebo for 2 weeks of treatment. The subjects were young (mean age 42 years), predominantly White and female. One month later, the outcome was assessed with quantitative health measures. No difference was observed between the two groups (Sawano et al. 2025). In a similar study design, adults with long‐term moderate to severe PASC from California were randomised for treatment with nirmatrelvir/ritonavir (*n* = 102) over 2 weeks or placebo (*n* = 53). Ten weeks later, there was no significant difference in six core PASC symptoms between the treatment and control group (Geng et al. 2024).

## 6 Other Antiviral Agents

### 6.1 Lopinavir

**FIGURE 8:**
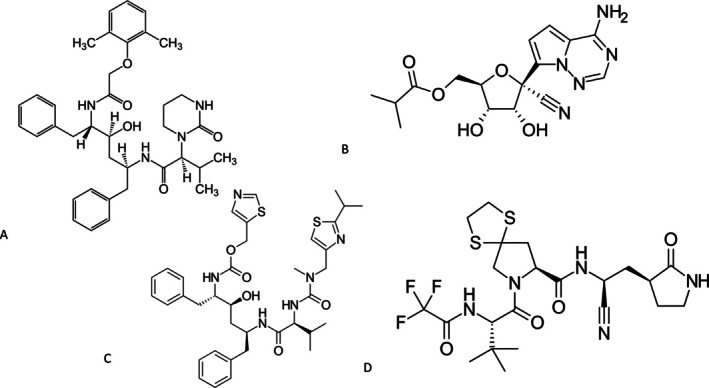
Further antivirals used in COVID‐19 clinical trials. Lopinavir (A), a 3CL protease inhibitor; Obeldesivir (B), an RNA‐dependent RNA polymerase inhibitor; Ritonavir (C), initially developed as an HIV protease inhibitor, it inhibits the liver enzyme cytochrome P450 and acts therefore as a pharmacological booster in combination with other antivirals such as simnotrelvir (D), a 3CL protease inhibitor. Figure credit: Jü, Innerstream from Wikipedia.

When screening more than 10,000 small chemical compounds for inhibitory activity against SARS‐CoV in Vero cells, Chinese researchers identified two anti‐human immunodeficiency virus (HIV) agents as the most promising candidates. One agent was an HIV entry blocker and the other lopinavir (Figure 8A), a 3CL protease inhibitor, displaying also anti‐SARS‐CoV activity in the sub‐micromolar concentration range (Wu et al. 2004). During the early phase of the pandemic (January 2020), Chinese clinicians tested lopinavir combined with ritonavir against standard care. The patients were hospitalised in Wuhan/China where the pandemic originated and suffered from severe COVID‐19 disease. The median interval time between symptom onset and randomisation was 13 days; treatment started thus late in comparison with later trials. Time to clinical improvement in the intention‐to‐treat population was slightly shorter in the lopinavir group (15 vs. 16 days in controls, primary endpoint). Mortality within 28 days was numerically lower in the lopinavir group (19% vs. 25% in control), which also showed a shorter stay in the intensive care (6 vs. 11 days in control), but the differences were statistically not significant. The decrease in viral load over time did not differ between both groups. Gastrointestinal adverse events were more common in the lopinavir group (Cao et al. 2020). In the WHO Solidarity trial enrolling severe COVID‐19 cases as indicated by a high in‐hospital death rate, 148 of 1399 patients receiving lopinavir and 146 of 1372 patients receiving its control died. Ventilation was initiated in 126 patients receiving lopinavir and in 121 receiving its control. Lopinavir was subsequently eliminated for futility from the Solidarity trial (WHO Solidarity Trial Consortium 2021, 2022).

### 6.2 Obeldesivir

Obeldesivir is an isobutyl‐ester prodrug of a cyano‐substituted adenosine analogue synthesised by Gilead scientists (Figure 8B). It can be given orally. Upon intestinal and liver passage, the isobutyryl rest is removed, the resulting metabolite circulates in the blood and is taken up by lung cells where it is phosphorylated to a nucleotide triphosphate by intracellular kinases. In monkeys infected with SARS‐CoV‐2, oral obeldesivir significantly reduced the viral load in the lungs compared to placebo‐treated monkeys (Mackman et al. 2023). A phase 3 multicenter clinical trial was conducted during 2023 in the US and Japan enrolling nearly 2000 subjects with mild‐to‐moderate COVID‐19 disease. Patients were randomised within 3 days after symptom onset on oral obeldesivir or placebo. Median time to COVID‐19 symptom alleviation (6 days) and symptom resolution (9.2 days) did not differ between the two groups. Post hoc analysis revealed a significantly shorter symptom duration in patients treated with obeldesivir within 1 day of symptom onset. No hospitalisations occurred in either group. The viral load was lower on days 3 and 5 in the obeldesivir group compared to controls (Ogbuagu et al. 2025). About 400 non‐hospitalised COVID‐19 patients were randomised on different continents on obeldesivir or placebo during 2023. Treatment started before the third day after symptom onset. Hospitalisation, the primary endpoint, and medically attended visit, the secondary endpoint, were only observed in 1 and 3 subjects, respectively. Median time to symptom alleviation was 2 days shorter in the obeldesivir group (7 vs. 9 days in control) and viral load was lower at day 5 in obeldesivir‐treated patients than in controls (Streinu‐Cercel et al. 2026).

### 6.3 Simnotrelvir

Chinese scientists optimised boceprevir, an approved small molecule antiviral drug that targets Hepatitis C virus NS3/4 A serine protease. This drug also showed weak inhibitory activity against SARS‐CoV‐2. Chemical modifications increased the specificity for the SARS‐CoV‐2 3CL protease by 50‐fold. Analysis of the crystal structure of SARS‐CoV‐2 3CL protease with this bound drug derivative led to further optimisation of the inhibitor, resulting in nanomolar inhibitory concentrations against various SARS‐CoV‐2 variants. In a mouse infection model, simnotrelvir decreased the viral lung and brain titers in a dose‐dependent way. In addition, it alleviated lung pathology and suppressed weight loss (Jiang et al. 2023). Simnotrelvir (Figure 8D) is approved in China for the treatment of mild to moderate COVID‐19. In a phase 2/3 clinical trial conducted at 35 sites in China, 1200 young and vaccinated COVID‐19 patients were treated with simnotrelvir plus ritonavir (Figure 8C) or placebo within 3 days after symptom onset. Median time to the resolution of symptoms was significantly shortened in simnotrelvir recipients (180 vs. 216 h in controls). Simnotrelvir accelerated the resolution of respiratory symptoms, but not of fever. The viral load in respiratory tract samples from treated patients was 10‐fold lower than in placebo recipients. More adverse events were observed in the simnotrelvir than the placebo group, but the events were not severe (Cao et al. 2024).

In a nationwide, multicenter, prospective, observational real‐world study at 42 sites in China 3500 subjects with mild to moderate COVID‐19 were randomised in 2023 to simnotrelvir plus ritonavir or placebo. As primary endpoint a 10‐fold lower percentage of hospitalisation was reported in simnotrelvir recipients (0.3% vs. 3.1% in controls), median time to symptom resolution was also shorter (8 vs. 10 days in controls) as well as time to viral clearance (6 vs. 7 days in controls) (Han et al. 2024).

## 7 Outlook

The medical, societal and economic impact of the COVID‐19 pandemic has led to massive governmental and industrial investments in SARS‐CoV‐2 vaccine and antiviral research. A large number of controlled clinical trials have been conducted that are only possible during a massive outbreak which turbocharged vaccine and antiviral development. It is now important to take learning lessons from these efforts for the future.

Reporting the outcome of individual published studies as done in the present overview necessarily creates a complex picture. In vitro results do not translate to results in animal infection models because of the increased complexity of drug interactions in a living mammalian animal compared to cell cultures. The failure of hydroxychloroquine in the early COVID‐19 antiviral drug trial is a vivid illustration that clinical trials cannot be based on cell culture assays alone. Antiviral small molecule screening programmes in cell culture can only be the first step in a larger drug search programme. Also, the animal models cannot be directly translated to human conditions since the pharmacological transformations of antiviral drugs differ in important ways between animals and humans. Even comparing the outcome of clinical trials in COVID‐19 patients is not straightforward. During the COVID‐19 pandemic, the infectious agent evolved over time. The successive SARS‐CoV‐2 variants differed in pathogenic potential, transmissibility and immunological properties. Therefore, clinical trials conducted at different phases of the pandemic or in different geographical areas are hard to compare. The human patients also changed in important respects. In early trials, the patients were immunologically naïve. In later tests, most patients were vaccinated or had immunological experience from natural infections. Then, clinical tests targeted patient populations that differed with respect to the clinical severity of disease, age, genetic background or comorbidity. Trial outcomes are likely to differ in patients displaying severe, moderate, or mild COVID‐19 disease or between inpatients and outpatients. In early trials, many patients displayed severe infections such that in‐hospital mortality could be taken as primary endpoints for clinical trials. In later trials when the combination of less virulent viral variants and increasing population immunity attenuated the clinical severity of the infection, hospitalisation instead of mortality had to be taken as clinical endpoint. Currently, the impact of antivirals on viral load decrease has to be used as read‐out of clinical trials because even hospitalisation criteria would need huge enrolment numbers to reach statistical significance.

There are further factors affecting the outcome which complicate the interpretation of results. An important parameter is when the antiviral drug has been given after symptom onset. For an acute infection, an early antiviral drug treatment is expected to have a greater effect than when antivirals are given later and the clinical symptoms are possibly dominated by immunopathology effects. Animal models suggest that the time window for an efficient antiviral application can be a few days. Early treatment is however difficult to realise in clinical trials. The situation for antivirals might be more favourable in chronic viral infections. Such a situation might be found in long Covid patients where some data suggest an underlying chronic infection (Peluso et al. 2024; Zuo et al. 2024). However, the pathogenesis of long Covid is only just beginning to unravel (Greenhalgh et al. 2024) and it is still too early to decide whether persistent SARS‐CoV‐2 is a passive passenger or a driver of the symptoms in long Covid (Al‐Aly 2025). Treatment of the acute COVID‐19 infection with antivirals seem to reduce the later occurrence of long covid symptoms but the effects were small. Treatment trials of long covid patients with antivirals are few and were so far not successful.

To extract a simple take home message on the utility of COVID‐19 antivirals from a literature review of individual published clinical studies is thus hard. One might therefore look for recent meta‐analyses extracting and formally comparing data from all available randomised, controlled trials (RCT) and real world (RW) studies. For remdesivir, such a meta‐analysis based on 4 RCT (25,000 patients) and 16 RW studies (with 1.3 mio participants) concluded that remdesivir significantly increased survival in the overall population (odds ratio 0.69, *p* = 0.001), and this effect was observed across SARS‐CoV‐2 variants and across different disease severity levels defined by supplementary oxygen need at hospitalisation. Rehospitalisation risk was also significantly reduced (Bartoletti et al. 2025). A Cochrane meta‐analysis including COVID‐19 remdesivir treatment data published until mid‐2022 concluded with moderate certainty that remdesivir probably makes little or no difference to all‐cause mortality in moderate to severe COVID‐19 (8 fewer deaths per 1000 treated patients), but decreases the risk of clinical worsening (hazard ratio 0.67). Remdesivir decreased the risk of hospitalisation in mild COVID‐19 (Grundeis et al. 2023). A Cochrane meta‐analysis of molnupiravir versus control associated little or no difference in mortality (rare in the analysed trials) and hospitalisation with treatment in outpatients, while data from inpatients were too heterogeneous to analyse (Tatz et al. 2025). A Cochrane meta‐analyses on nirmatrelvir plus ritonavir treatment suggested with low‐certainty evidence a risk reduction of all‐cause mortality and hospital admission or death in high‐risk, unvaccinated COVID‐19 outpatients infected with the Delta variant of SARS‐CoV‐2. Very low‐certainty evidence exists regarding the effect on all‐cause mortality and viral clearance in mildly to moderately Omicron‐infected inpatients (Reis et al. 2023). Another meta‐analysis compared a wide range of drug treatments in patients with mild or moderate COVID‐19. This study concluded with moderate certainty that only two drugs reduced hospital admission: nirmatrelvir‐ritonavir (25 fewer hospitalisations per 1000 treated patients) and remdesivir (21 fewer per 1000 treated). Molnupiravir and systemic corticosteroids may reduce hospital admission (low certainty). No antiviral had an effect on mortality, which is not surprising since death is unlike in mild to moderate COVID‐19. Compared with standard care, only lopinavir‐ritonavir increased adverse effects (Ibrahim et al. 2025).

There are practical problems with the application of approved COVID‐19 antivirals. Remdesivir has to be given by intravenous injection, which poses problems in low‐resource countries. Molnupiravir should not be given to young women for concerns about potential mutagenic effects if they should be pregnant. Nirmatrelvir in its combination with ritonavir causes important drug–drug interactions. Then there are cost considerations: a course of Paxlovid (trade name for nirmatrelvir/ritonavir) treatment costs in the EU more than 1000 euros. With such a price tag, an antiviral becomes unaffordable for the health systems of low and middle‐income countries. Pfizer has agreed with Medicines Patent Pool in Geneva to licence its intellectual property to generics makers in these countries at a price of US$ 25 per course. Non‐profit organisations have bought 100,000 courses for distribution, but could not meet demands (Ledford 2022).

Overall, it is fair to conclude that antivirals acting on the two viral enzymes RdRp and the main protease had only a modest effect on the course of the COVID‐19 pandemic. In view of the urgent need for efficient antivirals, ongoing research on antivirals interfering with other coronavirus proteins or different replication steps is important. The Nobel‐prize winning mRNA vaccines, and not antivirals, were the game changers in the COVID‐19 pandemic. It is not understandable why the US government wants to suppress the US$ 1.2 billion earmarked for mRNA vaccine research and development. This comes in parallel with a downgrading of the US Centres for Disease Control and Prevention and attacks on leading US research universities. Vaccine development, infection epidemiology and fundamental research are the best hopes to cope not only with the next infection emergency but to better human health in general. American science institutions are worldwide one of the most admired facets of American greatness in recent decades. Human health, including that of US citizens, will suffer when ideology wins over evidence gained by experiments, observations and reasoning.

## Author Contributions

**Harald Brüssow:** conceptualization, investigation, writing – original draft.

## Funding

The author has nothing to report.

## Conflicts of Interest

The author declares no conflicts of interest.

## Data Availability

Data sharing not applicable to this article as no datasets were generated or analysed during the current study.
